# Effectiveness of peer-delivered Problem Management Plus for Immigrants (PMP-I) in reducing stress, anxiety, and depressive symptoms among Bhutanese adults resettled in Massachusetts, United States: a pilot randomized controlled trial

**DOI:** 10.1017/S2045796026100742

**Published:** 2026-07-24

**Authors:** Kalpana Poudel-Tandukar, Holly Laws, Christopher R. Martell, Shan Rai, Razu Ramdam, Jerrold S. Meyer, Elizabeth R. Bertone-Johnson, Cynthia S. Jacelon, Steven D. Hollon

**Affiliations:** 1 Elaine Marieb College of Nursing, University of Massachusetts Amherst, Amherst, MA, USA; 2 Institute for Global Health, University of Massachusetts Amherst, Amherst, MA, USA; 3 Department of Psychological and Brain Sciences, College of Natural Sciences, University of Massachusetts Amherst, Amherst, MA, USA; 4 Bhutanese Christian Society of Western Massachusetts, Westfield, MA, USA; 5 Department of Biostatistics and Epidemiology, School of Public Health and Health Sciences, University of Massachusetts Amherst, Amherst, MA, USA; 6 Department of Psychology, Vanderbilt University, Nashville, TN, USA

**Keywords:** behaviour therapy, community mental health, controlled trial, depression, lay counsellors, refugees, strength-based intervention, stress

## Abstract

**Aims:**

Refugees bear the highest burden of mental health issues; however, they often underuse available mental health services. Interventions aimed at those with diagnosed conditions frequently do little to prevent the onset of disorders. Problem Management Plus for Immigrants (PMP-I) is a prevention intervention adapted from the World Health Organization’s Problem Management Plus (PMP). PMP-I includes psychoeducation, problem-solving, behavioural activation, social support and networking, and mind-body exercises. This pilot randomized controlled trial (RCT) aimed to evaluate the impact of PMP-I on mental, social and emotional well-being outcomes among Bhutanese refugees resettled in Massachusetts.

**Methods:**

Bhutanese individuals aged 18 or older who had resettled in Massachusetts and scored ≤14 on the Patient Health Questionnaire-9 (PHQ-9) were randomly assigned to two groups: PMP-I (*n* = 58 families) and a talk programme with a community support services pamphlet (*n* = 58 families). Trained community interventionists delivered five sessions of PMP-I to intervention participants in their family settings. Primary outcomes included scores on measures of stress (Cohen Perceived Stress Scale-10), and anxiety and depression (Hopkins Symptoms Checklist-25), assessed at baseline, 6-week and 3-month post-intervention. Secondary outcomes included hair cortisol, coping, coping self-efficacy, social support, social network, family conflict resolution and family satisfaction. Linear mixed-effects models were used to analyse differences in changes in outcomes between the intervention and control groups, adjusting for baseline scores on the primary outcomes, as well as age, duration of residence, marital status and history of chronic diseases.

**Results:**

All 232 participants recruited (116 families) were retained throughout the project. The intervention group evidenced a significantly greater decrease than the control group at both 6-week and 3-month post-intervention assessments on the primary outcomes of stress, anxiety, and depressive symptom scores. Similarly, the intervention group demonstrated significantly higher scores at both the 6-week and 3-month periods after the intervention compared to the control group on measures of coping, family conflict resolution, self-efficacy, family satisfaction and social networking with large effect sizes (Cohen’s *d* > 0.8, *p* < .01). Hair cortisol concentrations did not differ significantly between the intervention and control groups at either baseline or 3-month post-intervention.

**Conclusions:**

The PMP-I improved the mental, social and emotional well-being of Bhutanese adults (PHQ-9 score ≤ 14) resettled in Massachusetts. Peer-delivered, family-centred PMP-I offers a significant public health benefit; however, a large-scale, RCT involving diverse refugee groups is necessary to replicate its success nationwide and beyond.

## Introduction

Problem Management Plus (PMP) is a low-intensity psychological intervention developed by the World Health Organization (Dawson *et al.*, 2015; World Health Organization, 2016). PMP included four major evidence-based interventions: stress management through breathing exercises (Wahbeh *et al.*, 2008; de Manincor *et al.*, 2015; Falsafi, 2016; Prathikanti *et al.*, 2017), problem solving (Cuijpers *et al.*, 2018), behavioural activation (Cuijpers *et al.*, 2007; Veale, 2008; Mazzucchelli *et al.*, 2009; Martell *et al.*, 2010), and skills to strengthen social support for adults experiencing psychological distress, anxiety or depression. Lay-trained people can deliver PMP in either individual, group or web-based formats. PMP has demonstrated effective results in reducing mental health problems among non-refugee populations in Nepal (Sangraula *et al.*, 2020; Jordans *et al.*, 2021), Kenya (Bryant *et al.*, 2022) and Pakistan (Rahman *et al.*, 2016, 2019; Khan *et al.*, 2019; Hamdani *et al.*, 2021), and as well as among refugees resettled outside the United States (Jordan [Bryant *et al.*, 2022; de Graaff *et al.*, 2023], Netherland [de Graaff *et al.*, 2023] and Austria [Knefel *et al.*, 2022]) with psychological distress or problems. It also helps improve subjective well-being and quality of life among Venezuelan migrants, refugees and Colombian returnees in Colombia (Perera *et al.*, 2022).

However, the effectiveness of the PMP intervention in promoting positive adaptive strategies – such as coping, self-efficacy, family conflict resolution, family satisfaction, and networking – mechanisms identified as essential for mental health promotion – has yet to be explored. Studies (Rahman *et al.*, 2016, 2019; Khan *et al.*, 2019; Sangraula *et al.*, 2020; Hamdani *et al.*, 2021; Jordans *et al.*, 2021; Bryant *et al.*, 2022) have focused on the effect of PMP among individuals with moderate or severe psychological distress, anxiety or depressive symptoms. Still, its benefits among individuals with mild or moderate depressive symptoms, the refugee population, and in family settings are limited. To our knowledge, ours is the first family-based intervention trial assessing its effect on mental, social, and emotional health outcomes among refugees with mild or moderate depressive symptoms (score of ≤14 on the Patient Health Questionnaire-9 [PHQ-9]).

The burden of mental health problems is greater among refugees than in the general population. The systematic review found rates of 31.5% for depressive disorders and 31.46% for post-traumatic stress disorder among refugees (Blackmore *et al.*, 2020). The World Mental Surveys show the lifetime prevalence was 12% for depressive disorder and 3.9% for post-traumatic stress disorder among the general population (Kessler *et al.*, 2009). Refugees resettled in the United States have a high mental health burden of anxiety, depression and psychological distress (CDC, 2013; Marshall *et al.*, 2005; Poudel-Tandukar *et al.*, 2019, 2024, 2022b). It may be due to various socio-cultural and emotional stressors associated with adjusting to a new socio-cultural environment, characterized by limited socio-cultural and language skills, and a lack of culturally mediated and protective social support resources (LeMaster *et al.*, 2018; Kamimura *et al.*, 2021; Yamin *et al.*, 2021). The culturally tailored intervention components of PMP, such as psychoeducation, problem-solving, behavioural activation and social support, are expected to be promising in addressing the multiple socio-cultural and emotional stressors that affect mental, social and emotional health promotion. Thus, we have adapted PMP for use with immigrants (PMP-I) guided by community needs, with promising results. Our earlier small-scale Social and Emotional Well-being intervention that included managing stress and mind-body exercise, strengthening communication and social networking, problem-solving, and creating a healthy family environment, demonstrated a more than 50% reduction in prevalence of anxiety and depressive symptoms with a pre-post design in group (Poudel-Tandukar *et al.*, 2021) and family (Poudel-Tandukar *et al.*, 2022b) settings as delivered by community interventionists (CIs) among Bhutanese refugees resettled in Massachusetts.

PMP-I is a culturally tailored preventive intervention that includes psychoeducation, problem-solving, behavioural activation, social support (90-minute) and mind-body exercises (90-minute). Trained CIs held weekly in-home sessions for 5-week together with family members. We conducted a two-arm randomized controlled trial (RCT) comparing PMP-I with community support services (CSS) pamphlets talk programme in Bhutanese aged 18 or above who were resettled in Massachusetts and had a score of ≤14 on the PHQ-9. Outcomes were measured at baseline, 6-week, and 3-month post-intervention follow-ups. Our central hypothesis was that PMP-I would reduce perceived stress (Cohen *et al.*, 1983), anxiety and depressive symptoms (Derogatis *et al.*, 1974) (primary outcomes), hair cortisol levels (Meyer and Novak, 2012; Meyer *et al.*, 2014) (secondary outcome), and increase coping (Tobin, 2000), self-efficacy (Chesney *et al.*, 2006), family conflict resolution (Roskos *et al.*, 2010), family satisfaction (Olson, 2010), social support (Zimet *et al.*, 1988) and social networks (Lubben *et al.*, 2006) (targets) at follow-ups relative to CSS pamphlets talk group. The full details of the trial protocol have already been published (Poudel-Tandukar *et al.*, 2022a).

## Methods

### Trial design and setting

This two-arm, pilot, RCT was conducted among Bhutanese adults resettled in Massachusetts. About 90,000 Bhutanese people have been resettled in different states since 2008 (The Kathmandu Post, n.d.). The burden of mental health problems is high in this population nationwide (depression: 20%; suicide rate: 21.5 per 100,000) (Centers for Disease Control and Prevention (CDC), 2013) and in western Massachusetts (depression: 23.8%; anxiety: 34.5%) (Poudel-Tandukar *et al.*, 2019). The University of Massachusetts Amherst Institutional Review Board approved this study (Protocol ID: 1837). An Independent Safety Monitor with mental health expertise oversaw the safety of this clinical trial, and no adverse events were reported during the trial period. The details of adverse event definitions were reported in the published protocol (Poudel-Tandukar *et al.*, 2022a). This trial was prospectively registered on ClinicalTrials.gov (NCT04453709). This study was reported in accordance with the Consolidated Standards of Reporting Trials (CONSORT), a primary reporting guideline for RCTs.

### Participants

The inclusion criteria for participants were Bhutanese adults aged 18 or older (including parents and children) who had resettled in Massachusetts and had a PHQ-9 score ≤14, as we aimed to evaluate the intervention’s effect on preventing depression by reducing symptom scores in advance. Our trial adopted a preventive strategy, targeting individuals with PHQ-9 scores of 14 or lower, informed by the community’s needs and willingness to address mental health concerns early, before they escalate into full-blown disorders. Our trial works best as a prevention trial, reducing risk in the low-symptom group and reducing symptoms and preventing disorder onset in the moderate/near-threshold group. Our approach emphasizes early intervention and proactive mental health care, offering a more scalable and accessible way to reduce the overall mental health burden, moving beyond traditional treatment-focused trials.

Participants with diagnosed mental disorders or those taking psychiatric medications were excluded. We used the PHQ-9 scale as a screening tool to identify individuals with a score of 15 or higher and exclude them from the study. We did not screen for or systematically exclude other psychiatric disorders. The PHQ-9 is a reliable and valid measure of depression severity and can also be used to make criteria-based diagnoses of depressive disorders. A validation study reported that patients with major depression were 13.6 times more likely to have a score of 15 or greater (Kroenke *et al.*, 2001). We included participants with a PHQ-9 score ≤14, whose risk of having an undiagnosed mental disorder may be low. To minimize bias, we adjusted for baseline scores on stress, anxiety and depressive disorders, as well as for a history of chronic disease, in all analysis models. Written informed consent was obtained from each participant. We provided $25 per survey (totalling $75 for the baseline and two follow-up surveys), $5 per intervention session (totalling $25 for five sessions) and an exercise mat to each participant.

### Randomization

We calculated a sample size of 116 families (58 per intervention arm) with an equal probability of being randomized to each of our two intervention arms by accounting for the intra-correlation among family members of .10 and *α* = .05, with an 80% power to detect a standardized difference of ES = .30 between the intervention and control groups using Optimal Design (Raudenbush, 2011). We randomly assigned families in a 1:1 ratio to the intervention or control group, allocating 116 interested families (58 per group) using a random number table. For random allocation, 116 numbers were selected from the table, using a block size of 12 to maintain balanced allocation between the two arms throughout the trial, given the feasibility of recruiting families of a certain size at once to implement the programme. The principal investigator (PI) requested that an independent community member who had no information about the participants’ characteristics and was not involved in participant recruitment, intervention delivery, or outcomes assessment point to a number in the random number table with their eyes closed. Since the selected families totalled 116, we used only the first three digits of the selected number to index the first sampled family. Together, we selected the remaining numbers, moving down the column of the first selected number. The PI generated the random sequence by assigning the first (odd) attempt number to the intervention and the second (even) attempt number to the control. The PI assigned numbers to the families in the sequential order provided by the community research assistants (RAs), who were unaware of the participants’ characteristics at the time of assignment. The intervention assignment was kept hidden from participants, RAs, CIs and community field supervisors (FS) before participants were enrolled in the trial. RAs explained the study procedures, obtained informed consent and discussed measures to protect participants. They successfully collected survey data from all participants at pre-, post-, and 3-month post-intervention surveys and were unaware of the families’ group assignments. To prevent contamination, participants were encouraged not to discuss intervention content with any other community members or RAs to minimize potential community conflict arising from other community members’ interest in participating in the intervention beyond our capacity. A separate group of community members was hired to deliver the PMP-I and talk programme interventions and to conduct surveys. They were encouraged not to discuss their programme experience among themselves.

### Intervention

PMP-I is a multicomponent preventive intervention including psychoeducation, problem-solving, behavioural activation, social support and networking (90-minute), as well as mind-body exercises (breathing and yoga: 90-minute), delivered in a family setting once a week for five weeks. Guided by Lazarus and Folkman’s stress theory (Lazarus and Folkman, 1987) and Bandura’s self-efficacy theories (Bandura, 1986, 1997), PMP-I aims to reduce stress by developing skills for coping positively, seeking support, and exploring other life-skills opportunities that can build self-esteem and self-efficacy, thereby enabling adjustment to a new sociocultural environment. It consists of five modules, which are as follows:

1. *Managing stress* – Stress management education, practice exercises for self-management of stress, breathing exercises and mind-body exercises. The separate sessions on breathing exercises and yoga were delivered to the intervention group participants across all 5 weeks, including guidelines for breathing and mind-body exercises and three types of breathing exercises (week 1), a seated stretch exercise sequence with six poses (week 2), a hands-and-knees exercise sequence with four poses (week 3), a moving flow exercise sequence with 20 poses (week 4) and a cool-down exercise sequence with five poses (week 5).

1. *Managing problems* – Problem-solving strategies and practice exercises to break down the problem into manageable components, develop solutions and practice implementing those solutions.

1. *Behavioural activation* – Communication skills and practice exercises to identify pleasant activities and then plan a strategy to carry out those tasks.

1. *Strengthening social support* – Social support and networking skills, including practice exercises to identify, plan and carry out networking tasks.

1. *Staying well* – Practice exercises to create a supportive and healthy family environment.

CIs delivered PMP-I through face-to-face sessions, breathing and mind-body exercises and homework that involved practicing activities to rebuild skills. CIs were Bhutanese community members with at least a high school education and no formal mental health training. They were trained for 12 days, following the World Health Organization’s PMP Helpers’ Training Guide (World Health Organization, 2018), adapted for PMP-I by research team members led by a cognitive-behavioural-oriented clinical psychologist with expertise in behavioural interventions (CM). CIs were trained for 4 hours in breathing exercises and 16 hours in mind-body exercises by a licensed yoga trainer. They used an intervention manual and pictographs to deliver the intervention to participants and their interested family members. Guided by lessons learned from our previous small-scale pilot intervention (Poudel-Tandukar *et al.*, 2022b) and community consensus, an intervention manual was developed in very simple English and translated and explained by interventionists during delivery. We recruited a separate group of CIs to deliver the PMP-I intervention to the intervention group (*n* = 6). Six trained CIs delivered PMP-I to participants in the intervention group. The same CIs delivered all intervention sessions to the same participant in the intervention group. Two FS and CIs monitored intervention delivery fidelity using a session-by-session checklist and a standard supervision checklist developed by the World Health Organization (World Health Organization, 2018). A detailed description of the measures was published in the protocol (Poudel-Tandukar *et al.*, 2022a). All participants completed five intervention sessions and completed baseline and follow-up surveys.

For the control group, a separate team of three CIs conducted about an hour of structured talk sessions with participants, using CSS pamphlets each week for 5 weeks. The pamphlets listed the names, contact information and service details of local community and health organizations, providing community members with information about local health and life-skills development services. CIs provided information on services offered by two community-based organizations at each visit. The same CIs delivered all intervention sessions to the same participant in the control group. After completing their 3-month post-intervention assessment, all control group participants received the brief two-session PMP-I intervention.

### Measures

Guided by scientific evidence and consensus from the research team and community members, we selected stress, anxiety and depressive symptoms as primary outcomes to measure an intervention’s success because of the high burden of these mental health problems in this population nationwide (Centers for Disease Control and Prevention (CDC), 2013) (depression: 20%) and in western Massachusetts (Poudel-Tandukar *et al.*, 2019) (depression: 23.8%; anxiety: 34.5%). Other measures, such as coping, self-efficacy and social support (described below), were selected as secondary outcomes because they provide supporting evidence for the primary outcomes. We used these measures in our earlier small-scale Social and Emotional Well-being intervention with a pre-post design, delivered by CIs, in a group (Poudel-Tandukar *et al.*, 2021) and family (Poudel-Tandukar *et al.*, 2022b) settings. The intervention reduced scores on the primary outcomes and improved scores on the secondary outcomes among Bhutanese refugees resettled in Massachusetts.

### Primary outcomes

#### Anxiety and depression

The Hopkins Symptom Checklist-25 (HSCL-25) (Derogatis *et al.*, 1974) consists of a 10-item subscale for anxiety and a 15-item subscale for depression, which were used to measure anxiety and depression (never [0] to 4 very often [4]), respectively, during the past month ([Cronbach’s α] for anxiety [α = 0.91] and depression [α = 0.92]). Items were summed into a cumulative score, with higher scores indicating elevated anxiety and depressive symptoms.

#### Perceived stress

The 10-item Cohen Perceived Stress Scale (PSS; Cohen *et al.*, 1983) was used to measure psychological stress (never [0] to very often [4]) experienced during the past month (Cronbach’s α = 0.71). The scores for questions 4, 5, 7 and 8 that were positively worded were reversed as follows: 0 = 4, 1 = 3, 3 = 1 and 4 = 0. Items were summed to yield a cumulative score, with higher scores indicating greater stress.

### Secondary outcomes

#### Physiological stress

Hair cortisol levels (averaged hormone levels over the past 3-month), a biomarker of physiological stress, were measured using the enzyme-linked immunosorbent assay (ELISA) (Meyer and Novak, 2012; Meyer *et al.*, 2014). The hair samples were analysed either at the Hormone Assay Laboratory at the University of Massachusetts Amherst or at the HAIR Lab at the Yale Child Study Center. A sensitive and specific enzyme immunoassay (Arbor Assays) was used for the analysis. The assay had intra- and inter-assay coefficients of variation of <10%. The results for each sample were normalized by dividing by the sample weight and expressed as picograms per milligram (pg/mg). The distribution of cortisol concentrations was right-skewed, so the values were log-transformed before further statistical analysis.

#### Coping strategy

The coping strategies were measured (not at all [1] to very much [5]) using a 32-item Coping Strategies Inventory-Short Form (CSI-SF; Cronbach’s α = 0.95) (Tobin, 2000). Items were summed to yield a cumulative score, with higher scores indicating better coping skills.

#### Coping self-efficacy

A 26-item Coping Self-efficacy (Chesney *et al.*, 2006) scale was employed to assess self-efficacy in coping (11-point Likert-type scale ranging from cannot do at all [0] to [10]certain can do [10]) (Cronbach’s α = 0.97). Items were summed into a cumulative score, with higher scores indicating stronger self-efficacy skills.

#### Social support

The 12-item Multidimensional Scale of Perceived Social Support (MSPSS; Zimet *et al.*, 1988) was used to measure social support (strongly disagree [1] to strongly agree [5])(Cronbach’s α = 0.95). Items were summed to yield a cumulative score, with higher scores indicating stronger social support skills.

#### Social network

The 6-item Lubben Social Network Scale-Revised (LSNS-R) (Lubben and Gironda, 2003; Lubben *et al.*, 2006) was used to measure social networks (none [0] to 9 or more or always [5]) among family (Cronbach’s α = 0.92) and friends (Cronbach’s α = 0.92). The cross-cultural social ties were measured with three questions in a similar pattern (Cronbach’s α = 0.92). Items were summed to yield a cumulative score, with higher scores indicating stronger social networking skills.

#### Family conflict resolution

The 17-item Family Conflict Resolution scale (Roskos *et al.*, 2010) was used to measure (never [1] to always [7]) conflict management (Cronbach’s α = 0.92). Items were summed into a cumulative score, with higher scores indicating stronger conflict-resolution skills.

#### Family satisfaction

A 10-item family satisfaction scale (Olson, 2010) was used to measure satisfaction (very dissatisfied [1] to extremely satisfied [5]) (Cronbach’s α = 0.94). Items were summed into a cumulative score, with higher scores indicating greater family satisfaction.

### Other variables

Age and length of residence in the US were measured in years. Other variables were dichotomized, including gender (male vs. female), marital status (ever vs. never), education (ever vs. never been to school), currently employed (yes vs. no), alcohol (ever vs. never), smoking (ever vs. never), and history of any chronic diseases (yes vs. no).

## Data collection

We recruited six Bhutanese community members from across Massachusetts as RAs and trained them to screen and recruit participants, obtain informed consent, administer surveys, collect hair samples and report adverse events. Trained RAs distributed study information through formal and informal networks, including word-of-mouth, phone calls, and gatherings, and verified participants’ eligibility. They obtained written informed consent from each participant and collected survey data face-to-face in a private setting at their house at pre-, post- and 3-month post-intervention time points using validated, structured questionnaires. Each family member was interviewed separately and privately to protect their privacy and confidentiality. Participants’ confidentiality was maintained by using numerical codes instead of names in all recordings. The same research assistant interviewed the same participant at both the baseline and follow-up surveys.

Because the data collection and intervention were conducted in person during the COVID-19 pandemic, we followed an ‘environmental health and safety screening tools for human subjects research’ developed by our university to ensure the safety of trainers, participants and family members. Initially, field staff screened participants by telephone using university-developed screening tools before making a home visit. If a participant answered ‘yes’ to any of these questions, such as ‘travelled to another state or internationally in the last 14 days while COVID-19 was prevalent’, ‘has someone you are in close contact with been diagnosed with COVID-19 (household, daycare, etc.)’, ‘in close contact with someone who is sick with respiratory symptoms’ or ‘feeling any of these symptoms such as fever (temperature over 100.3 F), cough, difficulty in breathing/shortness of breath, muscle aches, fatigue, headaches, sore throat, runny nose, nausea, vomiting, diarrhoea, loss of appetite’, field staff postponed the visit and encouraged the participant to monitor their health and symptoms and to contact their physician if they developed or were experiencing symptoms that were consistent with COVID-19. Field staff (fully vaccinated) travelled in their private car to visit participants. During the home visit, staff and participants wore facemasks and maintained a distance of 6 feet throughout field activities. Activities were performed indoors or outdoors, depending on participants’ preferences and the space available in their homes. Field staff cleaned their hands with alcohol-based hand sanitizers containing at least 60 percent alcohol before and after interacting with participants and cleaned all research materials with disinfectant wipes before and after use.

## Data analysis

We used a model-building approach to calculate effect sizes: we selected variables guided by theory and prior research, fitted regression models by adding the most significant covariates, and then computed standardized metrics, including Cohen’s d and mean differences, to assess practical significance alongside statistical tests. Means, standard deviations, and percentile distributions were used to describe the continuous baseline variables, and frequencies and percentages to describe categorical variables. We compared baseline characteristics between the intervention and control groups using chi-square tests and t-tests, as appropriate. Indices that differed significantly between the two groups at baseline were treated as covariates in the primary analyses, including baseline scores on psychosocial measures, age (in years), duration in the United States (in years), marital status (yes/no) and history of chronic disease (yes/no). The effect size was calculated as the difference in the least squares means (adjusted for baseline) between the control and intervention groups from the mixed model, adjusted for covariates, divided by the raw pooled standard deviation.

Our primary analyses tested whether outcomes for participants in the PMP-I arm differed from those in the control arm. Mixed-effects modelling was used to compare outcomes across treatment arms while accounting for clustering of participants within families. We included a random intercept for family in the model, which accounts for both the nesting of time points within families and participants within families. All within-group variables were group-mean centred to isolate within-group effects from between-group variance. Continuous outcomes were analysed using hierarchical linear modelling, and dichotomous outcomes were analysed using multi-level generalized linear models with a Bernoulli distribution, which is appropriate for nonlinear binary outcomes (Raudenbush and Bryk, 2002). We collected data from two members for each of the 58 families in each intervention arm, and the correlation among family members’ responses was accounted for in the model.

Hierarchical or multi-level modelling is well-suited to these data, as it accounts for the interdependence of repeated measures within individuals as well as the clustering of members within families, and accommodates unbalanced designs (i.e., different family sizes) (Raudenbush and Bryk, 2002). All of our participants completed intervention sessions and survey questionnaires. All analyses were performed using SAS, version 9 (SAS Institute Inc, Cary, NC) using the PROC MIXED and PROC GLIMIXX commands.

## Results

**Figure 1.**
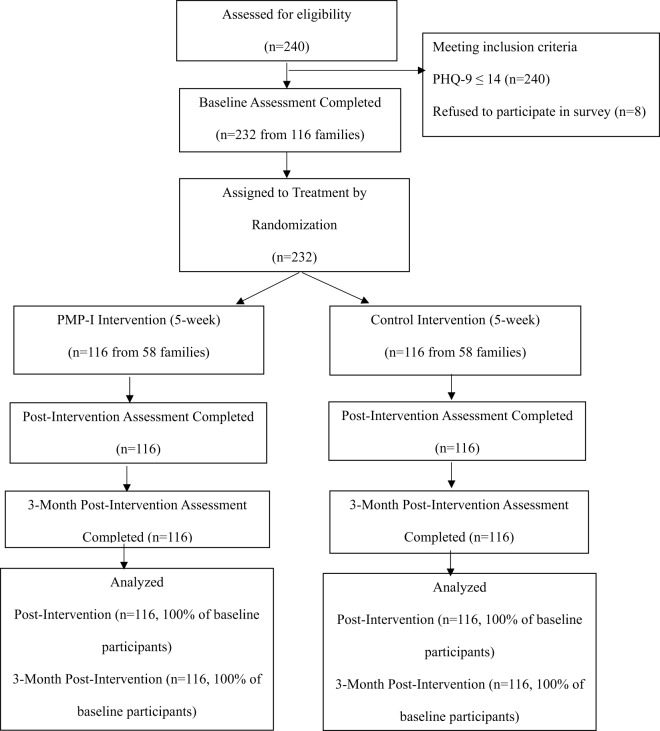
Flow chart. Module 1: long description.

We recruited the first participant on 17 August 2021, and completed a 3-month post-intervention survey on 15 November 2022. RAs screened 240 participants (Fig. 1). Eight participants refused to participate in the survey, and we included 232 participants from 116 families (two adults per family) in the study.

All recruited participants completed baseline and follow-up surveys and five intervention sessions. The mean age of the participants at baseline was 41.79 years (SD = 15.98), the average years living in the United States was 9.12 (SD = 2.10) and 44.39% of the participants were male, 85.34% were married, 50.43% were employed, 17.24% ever drank alcohol, 21.12% ever smoked, and 19.82% had a history of chronic diseases.

Tables 1 and 2 show group differences in characteristics and outcomes at the baseline. Groups differed significantly at baseline in terms of age, duration in the United States, marital status, history of chronic disease and primary and secondary outcomes. Thus, these differences were subsequently adjusted for in the mixed models.

**Table 1.** Characteristics of intervention and control groups at baseline Table 1 long description.

Characteristics	Intervention (*n* = 116)	Control (*n* = 116)	*p-value
Age, mean (SD)	43.60 (16.35)	39.98 (15.46)	0.08
Male, *n* (%)	52/116 (44.83)	51/116 (43.97)	0.89
Ever married, *n* (%)	104/116 (89.66)	94/116 (81.03)	0.06
Ever been to school, *n* (%)	63/116 (54.31)	62/116 (53.45)	0.89
Employed, *n* (%)	59/116 (50.86)	58/116 (50.0)	0.89
Ever drank alcohol, *n* (%)	23/116 (19.83)	17/116 (14.66)	0.23
Ever smoked, *n* (%)	25/116 (21.55)	24/116 (20.69)	0.87
Chronic disease, *n* (%)	31/116 (26.72)	15/116 (12.93)	<0.01
Duration in the United States in years, mean (SD)	9.45 (1.89)	8.77 (2.26)	0.01

* *p-*values were based on the Student *t*-test for continuous variables and the chi-square test for categorical variables.

**Table 2.** Group differences at baseline Table 2 long description.

Outcome measures	Intervention (*n* = 116)	Control (*n* = 116)	*p-value
**Baseline scores, mean (SD)**			
Stress	23.28 (3.70)	19.31 (4.87)	<.01
Anxiety	21.57 (3.25)	15.37 (5.08)	<.01
Depression	32.11 (4.89)	22.56 (6.24)	<.01
Coping	78.92 (15.26)	95.59 (19.53)	<.01
Family conflict resolution	69.05 (10.41)	85.27 (11.77)	<.01
Social support	53.75 (9.13)	65.50 (9.79)	<.01
Self-efficacy	145.6 (39.41)	168.9 (40.01)	<.01
Family satisfaction	32.08 (7.21)	38.30 (6.36)	<.01
Family networking	13.93 (4.31)	18.37 (5.58)	<.01
Friend networking	13.59 (4.44)	17.24 (5.57)	<.01
Community networking	4.78 (2.38)	6.65 (3.73)	<.01

* *p*-values were based on the Student *t*-test for continuous variables and chi-square test for categorical variables.

Table 3 presents the adjusted mean differences and effect sizes between the control and intervention groups at post-intervention. A multi-variate mixed model analysis was conducted to examine the post-intervention scores of the intervention group and the control group, controlling baseline scores and other significant covariates. Regarding the primary outcomes, at the post-intervention assessment, there were significant differences between control and intervention groups on stress (adjusted mean difference −8.40, 95% CI 7.62–9.18; *p* = < .0001, effect size (ES), 2.36), anxiety (adjusted mean difference 7.59, 95% CI 6.85–8.32; *p* = < .0001, ES, 2.08), and depression (adjusted mean difference 11.03, 95% CI 9.91 to 12.15; *p* = < .0001, ES, 2.21).

**Table 3.** Mixed model analysis of intervention effects on primary and secondary outcomes Table 3 long description.

		Descriptive statistics Estimated mean (S. E.)		Mixed model analysis		
Outcomes	Time point	PMP-I	Control	Difference in LS mean (95% CI)	*p*-value	Effect size
Stress	Baseline	22.09 (0.27)	20.49 (0.27)			
	6-week	11.67 (0.27)	20.07 (0.27)	8.40 (7.62, 9.18)	<.01	2.36
	3-month	12.35 (0.27)	19.68 (0.27)	7.33 (6.55, 8.11)	<.01	2.14
Anxiety	Baseline	19.44 (0.25)	17.51 (0.25)			
	6-week	9.22 (0.25)	16.81 (0.25)	7.59 (6.85, 8.32)	<.01	2.08
	3-month	8.78 (0.25)	15.97 (0.25)	7.18 (6.44, 7.92)	<.01	2.06
Depression	Baseline	28.96 (0.37)	25.07 (0.37)			
	6-week	14.04 (0.37)	25.08 (0.37)	11.03 (9.91, 12.15)	<.01	2.21
	3-month	13.45 (0.37)	23.90 (0.37)	10.45 (9.33, 11.58)	<.01	2.21
Hair Cortisol (pg/ml) (log-transformed)	Baseline	1.78 (0.07)	1.85 (0.08)			
	3-month	1.70 (0.08)	1.63 (0.09)	−0.07 (−0.34, 0.19)	0.57	0.07
Coping	Baseline	83.91 (1.27)	90.59 (1.27)			
	6-week	125.51 (1.27)	93.26 (1.27)	−32.25 (−35.88, −28.61)	<.01	2.03
	3-month	123.11 (1.27)	92.07 (1.27)	−31.03 (−34.67, −27.40)	<.01	2.05
Family Conflict Resolution	Baseline	74.81 (0.71)	79.51 (0.71)			
	6-week	101.75 (0.71)	79.76 (0.71)	−21.97 (−24.07, −19.88)	<.01	2.18
	3-month	104.11 (0.71)	81.39 (0.71)	−22.71 (−24.80, −20.62)	<.01	2.13
Social Support	Baseline	57.33 (0.75)	61.92 (0.75)			
	6-week	76.27 (0.75)	61.84 (0.75)	−14.42 (−16.61, −12.24)	<.01	1.63
	3-month	78.15 (0.75)	62.75 (0.75)	−15.40 (−17.58, −13.22)	<.01	1.60
Coping Self-Efficacy	Baseline	153.96 (2.07)	160.54 (2.07)			
	6-week	199.96 (2.07)	162.46 (2.07)	−37.5 (−43.35, −31.64)	<.01	1.00
	3-month	216.59 (2.07)	163.64 (2.07)	−52.94 (−58.8, −47.09)	<.01	1.58
Family Satisfaction	Baseline	33.71 (0.44)	36.67 (0.44)			
	6-week	46.24 (0.44)	39.40 (0.44)	−6.83 (−8.12, −5.55)	<.01	1.24
	3-month	48.48 (0.44)	40.98 (0.44)	−7.49 (−8.77, −6.20)	<.01	1.50
Family Networking	Baseline	15.13 (0.43)	17.17 (0.43)			
	6-week	23.32 (0.43)	16.85 (0.43)	−6.46 (−7.70, −5.23)	<.01	1.23
	3-month	23.82 (0.43)	16.88 (0.43)	−6.94 (−8.17, −5.71)	<.01	1.38
Friend Networking	Baseline	14.81 (0.39)	16.02 (0.39)			
	6-week	22.03 (0.39)	16.08 (0.39)	−5.94 (−7.07, −4.82)	<.01	1.08
	3-month	23.02 (0.39)	15.92 (0.39)	−7.10 (−8.22, −5.97)	<.01	1.38
Community Networking	Baseline	5.36 (0.24)	6.07 (0.24)			
	6-week	9.55 (0.24)	6.34 (0.24)	−3.21 (−3.91, −2.51)	<.01	0.95
	3-month	9.83 (0.24)	5.93 (0.24)	−3.89 (−4.59, −3.19)	<.01	1.29

Stress = 10-item Cohen Perceived Stress Scale (range 0–40, higher scores indicated elevated stress); Anxiety or Depressive Symptoms = 25-item Hopkins Symptoms Checklist (range 10–40, higher scores indicated elevated anxiety; range 15–60, higher scores indicated elevated depressive symptoms); Coping = 32-item Coping Strategies Inventory-Short Form Checklist (range 32–160, higher scores indicated good coping skills); Family Conflict Resolution = 17-item Family Conflict Resolution Scale (range 17–119, higher scores indicated good conflict resolution skills); Social Support = 12-item Multidimensional Scale of Perceived Social Support (range 12–60, higher scores indicated good social support skills); Coping Self-Efficacy = 26-item Coping Self-Efficacy Scale (range 0–260, higher scores indicated good self-efficacy skills); Family Satisfaction = 10-item Family Satisfaction Scale (range 10–50, higher scores indicated good family satisfaction); Social Network = 6-item Lubben Social Network Scale (range 0–30, higher scores indicated good family or friends networking skills; 3-item Community Networking Scale (range 0–15, higher scores indicated good community networking skills).

Effect size was calculated as the difference in the least squares means between the control and intervention groups from a mixed model, adjusted for baseline outcome scores, age, duration of stay in the United States, marital status and history of chronic disease, divided by the raw pooled standard deviation.

At the 3-month post-intervention assessment, there were still significant differences between control and intervention groups, though the effect sizes were slightly lower, on stress (adjusted mean difference 7.33, 95% CI 6.55–8.11; *p* = < .0001, ES, 2.14), anxiety (adjusted mean difference 7.18, 95% CI 6.44 to 7.92; *p* = < .0001, ES, 2.06), and depression (adjusted mean difference 10.45, 95% CI 9.33–11.58; *p* = < .0001, ES, 2.21). The ES at post-intervention was larger than at 3-month post-intervention but remained clinically meaningful at both time points. We found no significant differences in hair cortisol concentrations between the intervention and control groups at follow-up.

Regarding secondary outcomes, at the post-intervention assessment, there were significant differences between control and intervention groups on coping (adjusted mean difference −32.25, 95% CI −35.88 to −28.61; *p* = <.0001, ES, 2.03), family conflict resolution (adjusted mean difference −21.97, 95% CI −24.07 to −19.88; *p* = <.0001, ES, 2.18), social support (adjusted mean difference −14.42, 95% CI −16.61 to −12.24; *p* = <.0001, ES, 1.63), coping self-efficacy (adjusted mean difference −37.5, 95% CI −43.35 to −31.64; *p* = <.0001, ES, 1.00), family satisfaction (adjusted mean difference −6.83, 95% CI −8.12 to −5.55; *p* = <.0001, ES, 1.24), family networking (adjusted mean difference −6.46, 95% CI −7.70 to −5.23; *p* = <.0001, ES, 1.23) friend networking (adjusted mean difference −5.94, 95% CI −7.07 to −4.82; *p* = <.0001, ES, 1.08) and community networking (adjusted mean difference −3.21, 95% CI −3.91 to −2.51; *p* = <.0001, ES, 0.95).

At the 3-month post-intervention, differences still were significant between control and intervention groups on coping (adjusted mean difference −31.03, 95% CI −34.67 to −27.40; *p* = <.0001, ES, 2.05), family conflict resolution (adjusted mean difference −22.71, 95% CI −24.80 to −20.62; *p* = <.0001, ES, 2.13), social support (adjusted mean difference −15.40, 95% CI −17.58 to −13.22; *p* = <.0001, ES, 1.60), coping self-efficacy (adjusted mean difference −52.94, 95% CI −58.80 to −47.09; *p* = <.0001, ES, 1.58), family satisfaction (adjusted mean difference −7.49, 95% CI −8.77 to −6.20; *p* = <.0001, effect size, 1.50), family networking (adjusted mean difference −6.94, 95% CI −8.17 to −5.71; *p* = <.0001, ES, 1.38), friend networking (adjusted mean difference −7.10, 95% CI −8.22 to −5.97; *p* = <.0001, ES, 1.38), and community networking (adjusted mean difference −3.89, 95% CI −4.59 to −3.19; *p* = <.0001, ES, 1.29) (Table 3).

**Figure 2.**
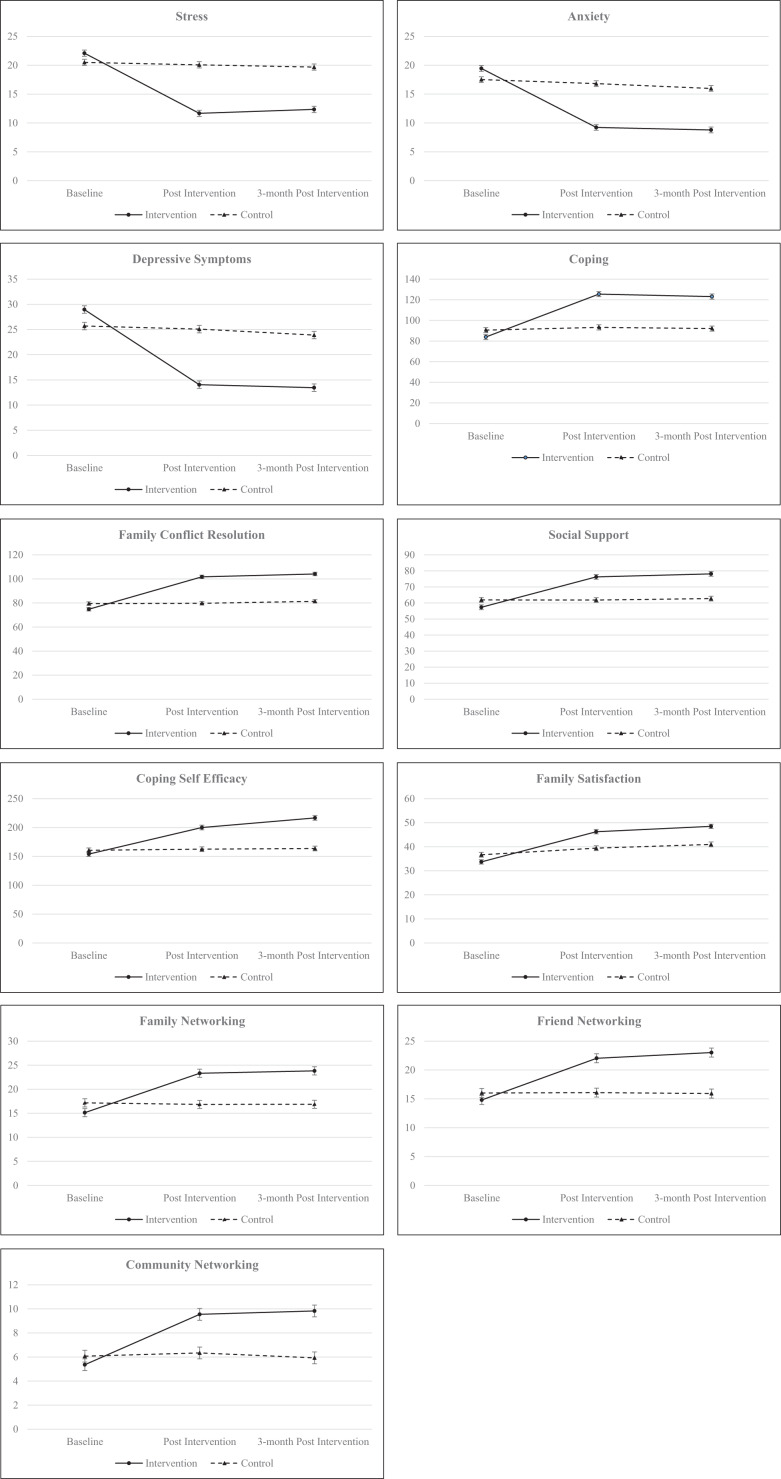
Means of primary and secondary outcomes with adjustment of baseline outcome scores and other covariates such as age (years), duration in the U.S. (years), ever married (yes/no), history of chronic disease (yes/no).

*Note*: Error bars indicate 95% confidence intervals

Figure 2 illustrates the adjusted mean scores for primary and secondary outcomes at baseline, 6-week and 3-month post-intervention. For the intervention group, the adjusted mean scores of primary outcomes decreased significantly from baseline to post-intervention and remained stable at the 3-month post-intervention point, except for hair cortisol concentration. The adjusted mean scores of secondary outcomes increased significantly from baseline to post-intervention and remained relatively stable at the 3-month post-intervention point. For the control group, the adjusted mean scores of primary and secondary outcomes remained largely unchanged from baseline to post-intervention and 3 months post-intervention.

## Discussion

Our pilot trial showed that PMP-I significantly lowered stress, anxiety and depression scores among Bhutanese adults in a non-clinical sample compared to the talk programme with an information-only control, with large effect sizes (Cohen’s *d* > 0.8) (Carson, 2012), at both post-intervention follow-ups, indicating that approximately 79% of the intervention group outperformed the control group average (Cohen, 1988). PMP-I also improved the average scores for coping, family conflict resolution, social support, self-efficacy, family satisfaction and networking with family, friends or community in the intervention group during follow-ups. The mental health symptom scores decreased, and coping and social support scores increased in the PMP-I condition but did not change in the control. To our knowledge, this is the first study to evaluate the impact of the PMP-I intervention, delivered by peer refugees, on reducing mental health symptoms and improving coping, self-efficacy, social networking and family satisfaction, which are considered key mechanisms for reducing anxiety and depressive symptoms among refugees without severe depressive symptoms who are resettled in the United States.

A unique finding of our trial is the significant large effects of peer-refugees delivered PMP-I on reducing stress, anxiety and depressive symptoms scores among refugees. We recruited community members from various areas and trained them as RAs, who then recruited participants and administered baseline and two follow-up surveys. Similarly, we trained community members as CIs, following the WHO PMP training guidelines. These CIs successfully delivered five intervention sessions to all participants and their interested family members in their native language, accommodating participants’ selected time frames. The intervention content was tailored to the participants’ needs during delivery. Participants were given the flexibility to choose the best options for each intervention module that worked for them and received necessary feedback the following week. Thus, the community engagement strategy throughout the research process, from the initial identification of needs to the design, implementation and evaluation of the intervention, played a significant role in the programme’s success.

Contrary to our target populations, in previous trials, PMP delivered by lay therapists produced a moderate effect size in reducing psychological distress among women who had experienced gender violence in Kenya (Bryant *et al.*, 2017), and anxiety and depression among routine patients from primary care centres with emotional distress in Pakistan (Rahman *et al.*, 2016). In one small trial among adult Syrian refugees (*N* = 60) in Amsterdam with elevated psychological distress and reduced psychosocial functioning, peer-refugee delivered PMP showed a moderate effect size in reducing anxiety and depressive symptoms (de Graaff *et al.*, 2020). The key finding that our study adds to existing literature is that PMP also helps to reduce stress, anxiety and depressive symptoms among refugees without severe depressive symptoms.

Our pilot trial also showed significant improvements in coping, family conflict resolution, social support, self-efficacy, family satisfaction and social networking, with strong effect sizes at post-intervention follow-ups in the intervention group. Importantly, we customized the practice plan for each intervention component, including problem-solving, behavioural activation, communication and social networking, to meet individual needs and promote practice within family settings. Interventionists followed the manual’s standard guidelines for each intervention component to identify feasible strategies tailored to each participant’s needs. For example, interventionists followed seven standard steps to develop a problem-solving practice plan: identifying the problem, prioritizing it, defining it, brainstorming solutions, choosing helpful, achievable ones and reviewing the plan. The problem-solving practice plan for the same problem, for example, a language barrier, differed among participants, such as learning five new English words per day, or engaging in frequent interaction with kids and grandkids at the dining table in English, or finding opportunities to interact more with native speakers.

Participants prepared for and practiced their weekly sessions to enhance desired coping and conflict-management skills. They were also encouraged to develop new or expand existing social networks within and outside their culture, depending on the issues they wanted to address. This flexibility and adaptation in the intervention content may be crucial for improving coping and self-efficacy among intervention group participants compared with the control group. Previous trials have shown moderate improvements in functioning and quality of life – covering aspects such as physical health, psychological well-being, social interactions and environmental factors – among Venezuelan migrants and refugees receiving psychological interventions in Ireland (Perera *et al.*, 2022). Our study adds a significant finding: the protective effect of PMP on coping, self-efficacy, conflict resolution and social networking skills in refugees without severe depressive symptoms.

The post-intervention improvements in coping, self-efficacy and networking skills persisted at the 3-month follow-up among participants in the intervention group in our trial. Participants likely can strengthen their skills by practicing more regularly over time, which will help them maintain their progress. Additionally, the presence of trained CIs whom the community trusts and shares the same language, lived experiences, and cultural lens as the community may provide strong cultural healing support to participants, helping them build their self-efficacy and networking skills. Studies have shown that the lay counsellors’ delivered psychotherapeutic interventions were effective in enhancing coping strategies and reducing stress disorder, physical ailments and social isolation (Fortuna *et al.*, 2019; Puschner *et al.*, 2019; Poudel-Tandukar *et al.*, 2022b), particularly among conflict-affected individuals (Neuner *et al.*, 2004). Moreover, other trials indicate that peers may promote social and emotional healing outcomes, such as self-efficacy, self-esteem, hope and social engagement, more effectively than clinical professionals (Davidson *et al.*, 2012). Although we lacked a direct comparison to clinical professionals, our controlled comparison was consistent with the notion that peers can provide interventions of value, as a lack of randomized control groups limited previous studies (Murray *et al.*, 2010).

A key strength of the study was the successful implementation of the programme and follow-up surveys, achieving 100% participant coverage at the intervention delivery, post-intervention, and 3-month post-intervention follow-ups, despite the challenges posed by the COVID-19 pandemic. Our trial demonstrated that CIs delivered a PMP-I that sustained protective effects on mental, social, and emotional well-being outcomes compared with a control group. The primary outcomes of this trial demonstrated at least a 10-point difference in stress, anxiety, and depressive symptoms scores, which is a highly significant effect among participants with a PHQ-9 score of 14 or below, serving as a preventive measure.

The current trial has some limitations, although community-based research has several strengths. First, we used the HSCL-25 to measure anxiety and depressive symptoms, which was validated with a clinical Diagnostic and Statistical Manual of Mental Disorders-4 (DSM-IV) diagnoses of major depressive disorder (APA, 1994), among refugees in Nepal (Shrestha *et al.*, 1998), and other countries (Hollifield *et al.*, 2002). The scale had high internal consistency (Cronbach’s *α*) for anxiety (Cronbach’s *α* = 0.91) and depression (Cronbach’s *α* = 0.92) in the current study, indicating a high degree of reliability. Although clinical diagnosis is the gold standard, this approach is not feasible in community-based studies. Second, we implemented our intervention during the COVID-19 pandemic, so it may have influenced the baseline mental health symptom burden in both the intervention and control groups. Therefore, any differential improvement in the intervention group over the control group is likely attributable to the intervention. It is possible that the stress induced by the pandemic increased initial scores in both conditions and amplified the overall effects of the intervention, but that is something that will have to be tested in subsequent replications.

Moreover, we used an ‘environmental health and safety screening tools for human subjects research’ developed by our university to ensure the safety of trainers, participants, and family members, as described above in the data collection section. The field team’s safety protocol was shared with all participants. Following these safety measures, participants raised no issues regarding the field staff’s home visits. Instead, they were glad to receive supportive interventions in their family settings and to work on stress management with their fellow interventionists, whom they trusted during their stressful period. However, the field staff needed to be flexible in conducting surveys and implementing intervention sessions while waiting for participants to recover from infections.

Third, we observed that randomization did not yield identical distributions of covariates and outcomes between groups, likely due to the small sample size or chance. For this reason, we treated baseline scores on psychosocial measures as covariates in regression models and controlled them in all outcome analyses, recognizing that this was the best that we could do given the limitations of randomization. Fourth, although the total compensation provided ($100) appears somewhat high, the fact that it was paid in increments spread over time ($25 per survey and $5 per intervention session) may have reduced its likelihood of influencing programme retention. Based on our experience working with this community for several years and feedback from participants, the field staff’s flexibility in accommodating participants’ preferred times for intervention sessions and surveys was an important factor in participants’ retention in the programme, alongside community members’ preferences for intervention content. Finally, we observed a relatively large effect size in our study; however, this was a preliminary result from a pilot trial and should be interpreted with caution, given the small sample size, which may lead to an overestimation of true effects due to sampling variability. Although our findings were encouraging, they would be even more compelling if replicated in a larger sample stratified by the primary outcomes prior to randomization.

The PMP-I had no effect on hair cortisol concentration, a biomarker of physiological stress, despite a significant reduction in perceived stress, as measured by the 10-item PSS. There are a few possible explanations for this apparent discrepancy. First, taken together, previously published studies have found no consistent relationship between hair cortisol and perceived stress (Gerber *et al.*, 2012; Stalder *et al.*, 2017; Prado-Gascó *et al.*, 2019). In many cases, including the present study, the two measures differ in their time scale. The perceived stress queries stressful feelings over the prior 30 days, whereas a 3-cm length of hair, which is the most common sample length in hair cortisol studies, contains cortisol that has accumulated over roughly the prior 3 months. In the present case, this time period of hormone accumulation was chosen because it provides a more reliable indicator of changes in chronic adrenocortical activity than briefer time periods. Nevertheless, it is possible that measuring cortisol in shorter (e.g., 1-cm) hair samples would have demonstrated a significant reduction in participants who underwent the PMP-I intervention. A second hypothesis is derived from evidence that changes in hair cortisol are more strongly related to objectively categorized major life events than to subjectively perceived stress (Karlén *et al.*, 2011; Gerber *et al.*, 2012; Li *et al.*, 2023). Perhaps the kinds of stressors experienced by the present Bhutanese refugee population and targeted by the PMP-I intervention do not fall within the category of events known to elicit activation of the hypothalamic-pituitary-adrenocortical axis. Indeed, because of the design of the clinical trial, it is unknown whether the Bhutanese refugees entered the trial with elevated cortisol levels in relation to a non-immigrant comparison group.

In conclusion, peer-delivered PMP-I was effective in improving mental, social, and emotional well-being among Bhutanese refugees (PHQ-9 score ≤ 14) resettled in Massachusetts. Engaging refugee families in designing, implementing, and evaluating a culturally competent psycho-socio mental health intervention was effective in reducing stress, anxiety, and depressive symptoms among Bhutanese refugees during the unique, difficult situation of the COVID-19 pandemic. Though our intervention appeared to provide a significant health benefit to the Bhutanese community, a large-scale controlled trial across diverse refugee groups, stratified by the primary outcome indices prior to randomization, is necessary to replicate its success nationwide and beyond.

## Supporting information

10.1017/S2045796026100742.sm001 Poudel-Tandukar et al. supplementary material Poudel-Tandukar et al. supplementary material

## Data Availability

Data analysed in this manuscript will be available upon request to the corresponding author.

## Supplementary material

The supplementary material for this article can be found at https://doi.org/10.1017/S2045796026100742.

## Module 1: Long description

The flowchart outlines the process of a study involving 240 participants assessed for eligibility. All met the inclusion criteria with a PHQ-9 score less than or equal to 14. Eight participants refused to participate, leaving 232 participants from 116 families for the baseline assessment. These participants were then assigned to treatment by randomization, resulting in two groups: PMP-I Intervention and Control Intervention, each consisting of 116 participants from 58 families. Both groups underwent a 5-week intervention. Post-intervention assessments were completed for all 116 participants in each group. A 3-month post-intervention assessment was also completed for all participants. The analysis showed that 100 percent of baseline participants completed both the post-intervention and 3-month post-intervention assessments in each group.

Navigate back to Module 1:.

## Table 1 Long description

The table compares baseline demographic and health characteristics between an intervention group and a control group, each with 116 participants, and reports p-values for group differences. Average age was 43.60 years in the intervention group and 39.98 years in the control group, with no statistically significant difference. The proportion male was similar, about 45 percent in both groups, and employment was also similar at about 51 percent in each group. Ever married was higher in the intervention group, about 90 percent versus 81 percent, but this difference was not statistically significant. School attendance, alcohol use, and smoking history were comparable between groups and not statistically significant. Chronic disease was more common in the intervention group, 31 of 116 compared with 15 of 116, and this difference was statistically significant. Time living in the United States was slightly longer in the intervention group, 9.45 years versus 8.77 years, and this difference was statistically significant. Overall, most baseline characteristics were balanced, with notable differences for chronic disease and years in the United States.

Navigate back to Table 1.

## Table 2 Long description

The table compares baseline mean scores with standard deviations for 11 psychosocial outcomes between an intervention group and a control group, with 116 participants in each. The intervention group started with higher symptom scores: stress 23.28 versus 19.31, anxiety 21.57 versus 15.37, and depression 32.11 versus 22.56. In contrast, the intervention group had lower resource and relationship scores, including coping 78.92 versus 95.59, family conflict resolution 69.05 versus 85.27, and social support 53.75 versus 65.50. Self-efficacy was also lower in the intervention group, 145.6 versus 168.9, and family satisfaction was 32.08 versus 38.30. Networking measures were lower for the intervention group across family, friend, and community domains: 13.93 versus 18.37, 13.59 versus 17.24, and 4.78 versus 6.65. Every outcome shows a statistically significant difference between groups, with p-values reported as less than point zero one. These results indicate the groups were not equivalent at baseline, so later outcome comparisons should account for these starting differences.

Navigate back to Table 2.

## Table 3 Long description

The table reports adjusted mixed model results comparing an intervention group (PMP-I) with a control group on mental health, coping, family and social outcomes at baseline, 6 weeks, and 3 months, using estimated means with standard errors. For stress, anxiety, and depression, both groups start with higher scores in the intervention group at baseline, but by 6 weeks and 3 months the intervention group shows markedly lower symptom scores than controls, with very large effect sizes and p-values below 0.01. Stress drops to about 11 to 12 in the intervention group versus about 20 in controls at both follow-ups; anxiety drops to about 9 versus about 16; depression drops to about 13 to 14 versus about 24 to 25. For positive functioning measures where higher scores indicate better outcomes, the intervention group improves substantially more than controls at 6 weeks and 3 months, including coping, family conflict resolution, social support, coping self-efficacy, family satisfaction, and family, friend, and community networking; effect sizes are generally large, roughly around 1 to above 2. Hair cortisol at 3 months shows little difference between groups, with a small effect size and a non-significant p-value. Differences are adjusted for baseline scores and participant characteristics, so results reflect modeled group differences rather than raw change alone.

Navigate back to Table 3.
